# The influence of blood on the human gut microbiome

**DOI:** 10.1186/s12866-020-01724-8

**Published:** 2020-03-03

**Authors:** Thierry Chénard, Mandy Malick, Jean Dubé, Eric Massé

**Affiliations:** Department of Biochemistry, Université de Sherbrooke, 3201 Jean-Mignault Street, Sherbrooke, Sherbrooke, QC J1E 4K8 Canada

**Keywords:** Gut microbiota, Colorectal cancer, Blood, 16S rRNA gene amplicon sequencing, Bacteria, Taxonomic biomarkers, LefSe, iFOBT testing

## Abstract

**Background:**

Colorectal cancer (CRC) is one of the prevailing causes of cancer mortality in the world. A common screening test for CRC is based on the human hemoglobin immunochemical based fecal occult blood test (iFOBT), which consists in the detection of blood in the patient’s stool. In addition to iFOBT, recent studies support the use of the gut microbiome as a biomarker for CRC prediction. However, these studies did not take into account the effect of blood itself on the microbiome composition, independently of CRC. Therefore, we investigated the microbiome of patients undergoing the iFOBT screening in order to determine the effect of blood alone. Our cohort consisted of patients who had no blood in their stools (*n* = 265) or did have blood but no underlying precancerous or cancerous lesions (*n* = 235). We also identified bacterial taxa specifically associated with the presence of blood in stools.

**Results:**

We observed significant differences in the intestinal bacterial composition that could be solely caused by the presence of blood in stools. More precisely, we identified 12 bacterial species showing significant differences in abundance between both our study groups. These species, *Bacteroides uniformis, Collinsella aerofaciens*, *Eggerthella lenta* and *Clostridium symbiosum* demonstrated increased abundance in the presence of blood. In contrast, the species *Prevotella copri*, *Coprococcus eutactus* and *catus*, *Faecalibacterium prausnitzii*, *Roseburia faecis*, *Blautia obeum*, *Gemmiger formicilis* and *Clostridium celatum* showed decreased abundance in patients with blood in their stools. Notably, we found multiple taxa that were reported in previous studies linking microbiome composition and diseases.

**Conclusions:**

We show that, in the absence of disease, blood in the stools has a major influence on the composition of the microbiome. Our data suggest that blood itself should be taken into consideration when investigating the microbiome signatures of intestinal diseases.

## Background

The development of various diseases such as type 2 diabetes, colorectal cancer (CRC), inflammatory bowel diseases and even depression, which is seemingly unrelated to the gut, has been discussed as being linked to the composition of the intestinal microbiome [1–9]. For example, *Fusobacterium nucleatum* has been identified as playing multiple roles in the development of CRC or its treatment [10], such as augmenting CRC cell proliferation [11] or increasing resistance to chemotherapy [12]. Moreover, enterotoxigenic *Bacteroides fragilis* [13] and polyketide synthase-producing *Escherichia coli* [14] have also been reported to promote colorectal cancer by producing DNA damaging molecules such as colobactin [15]. Remarkably, even though the microbiome and human health are tightly intertwined, the microbiome composition remains underused as a biomarker of disease progression or in the diagnosis process.

CRC is one of the three most common forms of cancer in both males and females [16]. The development of CRC starts with the formation of precancerous, mostly hyperplasic, polyps in the colon that eventually accumulate mutations until they form invasive malignant tumors [17]. It is critical to diagnose the development of the precancerous polyps early since relative survival rate drops from 90 to 65% after 5 years if the diagnosis is established once the tumors have become invasive [16].

To help detect early presence of CRC, the province of Québec (Canada) instated a province-wide CRC screening for patients over 50 years of age, a population with higher risks of developing CRC. This screening specifically uses the immunochemical fecal occult blood test (iFOBT) [18] to identify patients at high risks of having either precancerous polyps or colon cancer due to the presence of blood in their stools [19]. However, typical results from iFOBT screening exhibit a false-positive rate around 40% [20], which represents more than ten-thousand patients yearly, only in Québec, that are requested to undergo colonoscopy. In the group of false-positive patients, the presence of blood could be due to other minor problems such as inflammation or hemorrhoids. This high level of false-positive results have prompted many groups to investigate alternatives to iFOBT testing, such as specific intestinal microbiome signatures found in conjunction with colonic lesions [21–23].

Even though many reports have linked dysbiosis of the gut microbiome to the development of colon cancer and other intestinal diseases, none of these reports have, to our knowledge, investigated the effect of intestinal blood on the composition of the microbiome. Most of these studies compared healthy individuals to those that had developed intestinal lesions with no mention of the possible effect of the presence of blood in stool [22–25]. We hypothesized that the presence of blood in stools without any underlying intestinal lesions or polyps will affect the composition of the microbiome.

To address whether blood alone might influence microbiome analyses pertaining to bowel diseases, we sequenced the 16S rRNA gene from stool samples remaining after the iFOBT test of patients participating in Quebec’s province-wide CRC screening. None of the participants was suffering from CRC or any other important bowel diseases. The sequencing data was used to identify bacterial populations that had significant differences in abundance related to the presence or absence of blood in stools. Our analysis indicated that presence of blood in stools of patients without lesions (false-positive) has a major impact on the microbiome composition.

## Results

### Patient population

Our cohort is composed of 500 patients separated in two groups: 265 patients who had negative iFOBT results (patients negative for blood) and 235 who had positive iFOBT results (patients positive for blood) but whose colonoscopy did not reveal any trace of precancerous polyps or cancerous lesions (false positive). This study’s population were mostly recruited from patients undergoing Quebec’s province wide CRC screening in people aged 50 and over. The average age of our participants is around 61 and 62 years in patient groups with and without blood in their stool, respectively. The men/women split of both group is around 50/50. More information is available in Table 1.

**Table 1:** Patient group characteristics

	Patients without blood	Patients with blood
Number of patients	265	235
Age	61 ± 10	62 ± 13
Sex (M/F)	124/142	119/117

### Alpha and Beta diversity

**Fig. 1:**
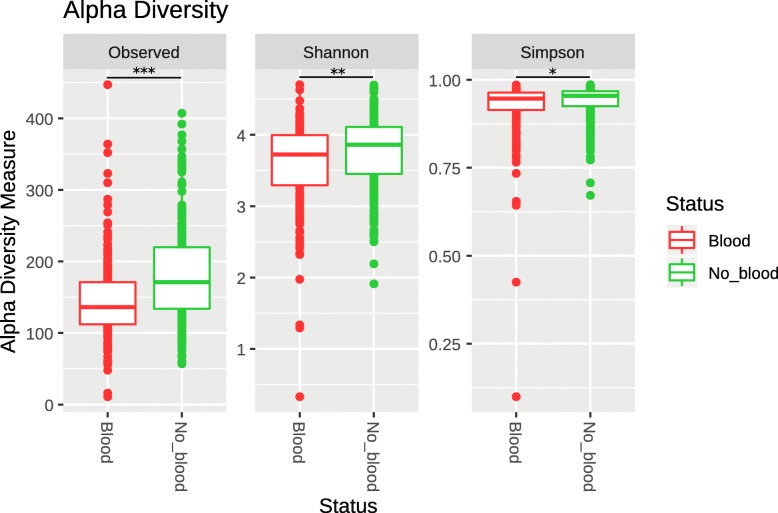
Various alpha-diversity measures (Observed OTUs, Shannon diversity index and the Simpson’s index) in all participants separated between both groups. In red, patients who had blood in their stool and in green patients who did not. *p-value< 0.05, **p-value< 0.005, ***p-value< 0.0005

**Fig. 2:**
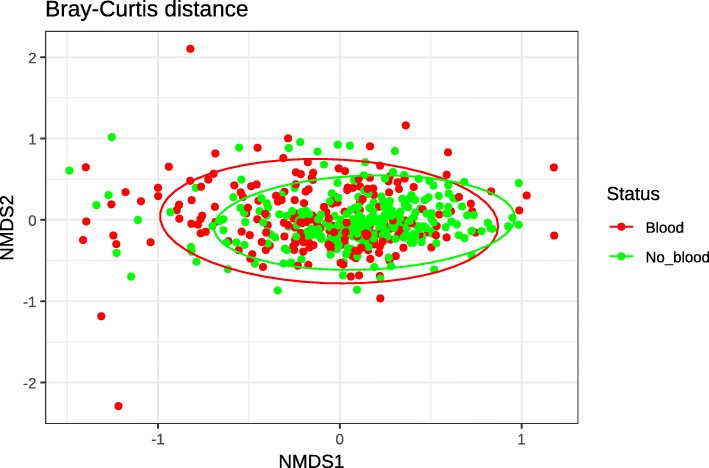
Visualization of the Bray-Curtis distance between samples in our study with ellipses representing the 95% confidence interval of each group. In red, patients who had blood in their stool and in green, patients who did not

We calculated 3 different measures of alpha diversity (observed operational taxonomic units (OTUs), Shannon diversity index [26] and Simpson’s index [27]) and compared their distribution to determine if there was a difference in the richness or evenness of the samples between both groups of patients. Patients without blood in their stools had significantly higher OTU count, evenness and diversity when compared to their counterparts who had blood. These data possibly suggest that the presence of intestinal blood negatively affects the overall composition of the microbiome (Fig. 1). In terms of beta-diversity, calculating the Bray-Curtis distance [28] between samples and plotting them shows no increased similarity between samples from the same group compared to samples of the opposing group (Fig. 2).

### Differently abundant taxa between patients with and without blood in their stool

**Fig. 3:**
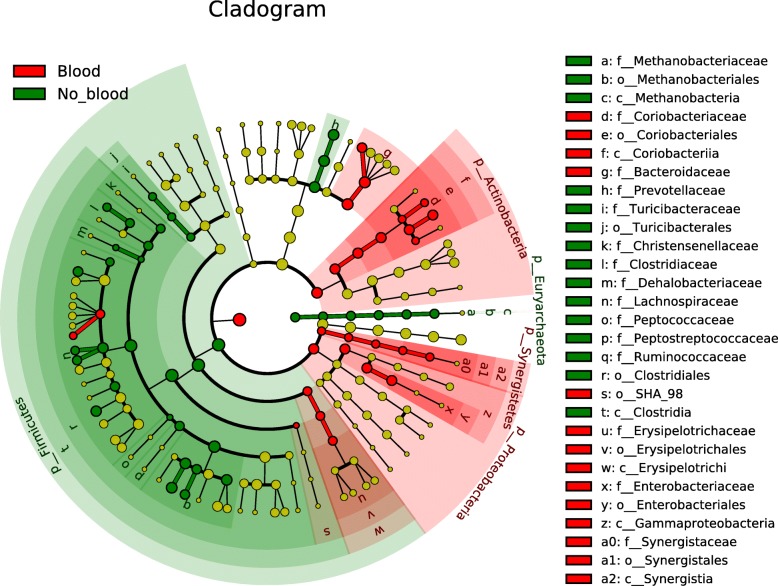
A circular phylogenetic tree (cladogram) showing the taxa differing between both groups. In red, taxa that have higher abundance in patients with blood and in green, taxa that have higher abundance in patients without blood. The letter in front of the taxon represent the taxonomy level. p: phylum, c: class, o: order, f: family

**Fig. 4:**
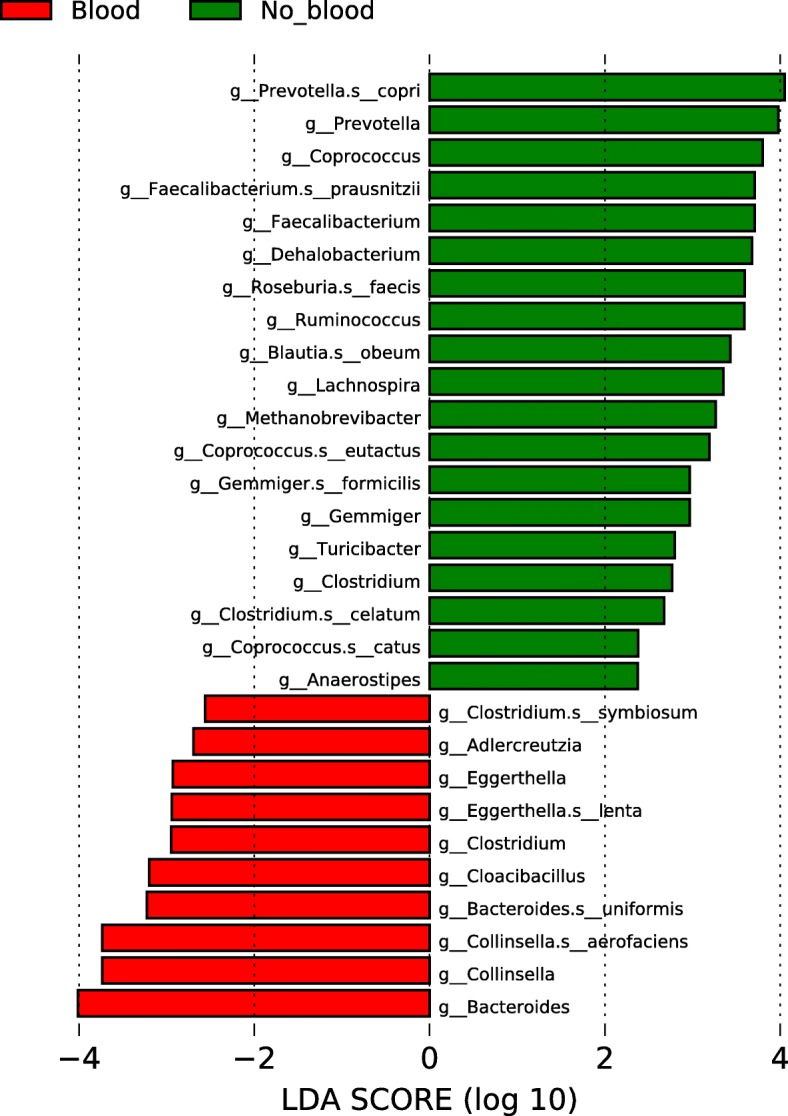
Graph showing the specific linear discrimination scores for the species and genera that had an absolute score over 2. In red, taxa that have higher abundance in patients with blood in their samples and in green, taxa that have higher abundance in patients without blood. The letter in front of the taxon represent the taxonomy level. g: genus, s: species

Using LEfSe (Linear discriminant analysis effect size) [29], we were able to identify taxa whose abundance differed significantly between participants who did not have blood in their stool and their counterpart who did (Figs. 3 and 4). These results indicate that differences can be identified at all taxonomic levels from the phylum to the species and that they spread through all the phyla present in our analysis except for the Synergistetes and Verrucomicrobia phyla. The taxa with higher abundance in participants without blood are part of the Firmicutes and Bacteroidetes phylum and the only Archea phylum in our analysis (Euryarchaeota). In addition, the taxa that were higher in the stools of patients with blood are part of the Bacteroidetes, Actinobacteria, Synergistetes and Proteobacteria phyla as well as some taxa in the Firmicutes. Similar differences were present in all taxonomic levels with 12 specific species and 17 genera having different abundance between both groups (Fig. 4). *Bacteroides uniformis, Collinsella aerofaciens*, *Eggerthella lenta* and *Clostridium symbiosum* are four bacterial species with increased abundance in our population with intestinal blood. On the opposing side, *Prevotella copri*, *Coprococcus eutactus* and *catus*, *Faecalibacterium prausnitzii*, *Roseburia faecis*, *Blautia obeum*, *Gemmiger formicilis* and *Clostridium celatum* are the bacterial species that had decreased abundance in patients with blood in their stool.

## Discussion

### Bacterial genera identified in our analysis

Most of the bacterial species whose abundance differed between patients with and without blood in their stool have been previously studied in various context. For instance, *Bacteroides uniformis,* which shows increased abundance in the presence of blood, has a specific subspecies (CECT 7771) that was previously shown to improve the metabolic and immunological dysfunction associated with obesity when administered to mice fed with a high-fat diet [30]. This bacterium is also currently investigated as a potential probiotic [31]. *B. uniformis* subspecies CECT 7771 was also shown to grow by using a variety of carbon sources such as insulin, pectin and wheat bran extract and produce butyrate and gamma-aminobutyric acid [32], which could help strengthen the gut barrier [33] and serve as a potential actor in the gut-brain axis [34]. *Collinsella aerofaciens*, a bacteria of the Actinobacteria phylum with increased abundance in patients with blood in their stools, also possesses a specific subspecies capable of butyrate production [35]. *C. aerofaciens* and *Eggerthella lenta,* another bacteria of the phylum Actinobacteria which had increased abundance in presence of blood, have also been reported as increasing gut permeability and lowering epithelial integrity in arthritis models [36, 37]. *E. lenta* bacteremia have also been reported as being more frequent but still rare in patients with advanced gastrointestinal or gynecological cancers [38]. Notably, some strains of *E. lenta* are capable of oxidizing bile acids, which potentially prevents the production of cancer-promoting secondary bile acids such as chenodeoxycholic acid [39]. Finally, *Clostridium symbiosum* from the phylum Firmicutes was more abundant in presence of blood. Importantly, *C. symbiosum* abundance was previously investigated as a potential biomarker for the early detection of CRC [40] and has been rarely reported to cause bacteremia in patients with colon [41] or ovarian cancers [42].

*Prevotella copri,* a member of the phylum Bacteroidetes, showing reduced abundance in presence of blood, is a predominant species in many people and is associated with a reduction of *Bacteroides* species as well as Lachnospiraceae and Group XIV Clostridia, which are usually described as favorable to intestinal health [43]. An increase in *P. copri* abundance has been reported as improving the fermentation of complex polysaccharides [44] but has also been correlated to increased risks of developing arthritis [45]. All the other bacteria we identified as having a reduced abundance in the presence of blood are part of the phylum Firmicutes. *Faecalibacterium prausnitzii* is considered one of the main butyrate producer found in the gut [46]. High-fat fed mice treated with oral *F. prausnitzii* had less adipose tissue inflammation and increased insulin sensitivity associated with lower hepatic fat content [47]. Moreover, *F. prausnitzii* depletion has been reported in multiple bowel disorders such as Crohn’s disease, colitis as well as colon cancer [48]. *Coprococcus eutactus* and *catus, Roseburia faecis* and *Blautia obeum* are genera with little known about them*. C. catus* was described to produce both propionate and butyrate, two metabolites beneficial for human health [49] and *C. eutactus* abundance was reported as reduced in patients with irritable bowel syndrome [50] and Parkinson’s disease [51]. *R. faecis* also produces butyrate and is able to produce an inhibitory substance against *Bacillus subtilis* [52]*. B. obeum* is capable of hydrolyzing bile salts [53] and produces a lantibiotic peptide effective against multiple *Clostridium* species [54, 55]. Finally, *Clostridium celatum* and *Gemmiger formicilis* have no known association to non-infectious human diseases nor are they known to produce metabolites of interest.

### Potential effect of blood on the microbiome being attributed to diseases in previous studies

To show the possible importance of considering the presence of blood in stools of patients, we compared the taxa whose abundance vary between our study and four published studies comparing the microbiome of patients suffering from CRC, Crohn’s disease, multiple sclerosis, ulcerative colitis and rheumatoid arthritis [22–25] (Table 2). All the reported studies have precision that maximally reached the genus level and some of them had different OTUs for the same genus with variations in different directions making it harder to compare precisely. For example, Zackular et al. reported OTUs associated with the *Clostridium* genus both increased and decreased in patients with adenomas while our analysis showed increased levels of *Clotridium* and more precisely *C. symbiosum* but a decreased level of *C. celatum*, which could reflect their finding but with higher resolution. Additionally, except for the study by Gevers et al. [25]*,* all the previously published reports used around 100 patients separated between their specific conditions. Finally, both analyses pertaining to CRC had the most differences in the variation of common bacteria with our analysis. This possibly indicates that cancerous lesions are associated with more distinct variations of the microbiome than blood itself when comparing it to other diseases such as Crohn’s or colitis.

**Table 2:** Taxa from published studies on various diseases that we also found in our study

	Direction of variation in abundance in article	Direction of variation in abundance in our analysis (Blood vs Control)
**Flemer et al. (2017)**		
Colorectal cancer vs control		
*Bacteroides*	↑	↑
*Roseburia*	↑	↓(*faecis*)
*Ruminococcus*	↑	↓
**Zackular et al. (2014)**		
Adenoma vs control		
*Ruminococcaceae*	↑	↓
*Clostridium*	↑/↓^a^	↑
*Bacteroides*	↓	↑
*Lachnospiraceae*	↓	↓
*Clostridiales*	↓	↓
Carcinoma vs control		
*Lachnospiriaceae*	↑/↓^a^	↓
*Enterobacteriaceae*	↑	↑
*Bacteroides*	↓	↑
*Clostridiales*	↓	↓
**Gevers et al. (2014)**		
Crohn’s disease vs control		
*Enterobacteriaceae*	↑	↑
*Bacteroides*	↓	↓
*Clostridiales*	↓	↓
*Erysipelotrichaceae*	↓	↑
*Faecalibacterium*	↓	↓
*Roseburia*	↓	↓(*feacis*)
*Blautia*	↓	↓(*obeum*)
*Ruminococcus*	↓	↓
*Coprococcus*	↓	↓
**Forbes et al. (2018)**		
All diseases vs control		
*Eggerthella*	↑	↑
*Clostridium*	↑	↑
*Gemmiger*	↓	↓
*Lachnospira*	↓	↓
Crohn’s disease vs control		
*Blautia*	↑	↓(*obeum*)
Ulcerative colitis		
*Clostridium*	↑/↓^a^	↑
*Coprococcus*	↓	↓
*Ruminococcus*	↓	↓
Multiple sclerosis vs control		
*Erysipelotrichaceae*	↑	↑
Rheumatoid arthritis vs control		
*Roseburia*	↓	↓(*faecis*)

↓ = decreased abundance in altered state. ↑ = increased abundance in altered state. () = we found a significant variation in the specific species from the genera but not in the genera itself. ^a^ = the authors found augmentation and diminution in abundance of OTUs associated with the same genus

## Conclusion

In conclusion, we have shown using a cohort of 500 patients that the presence of blood in stools has a major effect on the composition of the intestinal microbiome with more than 10 bacterial species having significant variations in their abundance. Furthermore, we showed that multiple taxa previously associated with the presence of diseases in other studies could be associated with the presence of intestinal blood. The results of our study indicate that the presence of blood should be considered as an important parameter when studying intestinal diseases and their related microbiomes.

## Methods

### Patient recruitment and sample collection

We obtained patient stool samples remaining after the iFOBT performed at the CIUSSS de l’Estrie - CHUS. Those samples were collected using the OC-Auto FIT collection kit (Polymedco) and kept at − 80 °C until use. For this experiment, we used samples with negative iFOBT results and those from subjects with positive iFOBT results but for whom the subsequent colonoscopy revealed no trace of neither precancerous polyps nor cancerous lesions or other severe bowel diseases. A positive iFOBT result is currently defined by an amount of blood in the stool over 175 ng/ml (defined by the *Ministère de la Santé et des Services Sociaux-MSSS*). Since we only had access to the patients’ medical file, and not the patients themselves, we were able to obtain patients sex and age. However, additional commonly used personal information such as BMI, ethnicity, blood glucose levels or smoking status were not available for most of the participants and could not be used in our analysis. We excluded, from our study, patients who suffered from other severe diseases such as bacterial infections or any other cancers. This study was approved by the research ethics committee of the CIUSSS de l’Estrie - CHUS (#2016–1197) and the consent of all participants was obtained via a reverse consent form.

### DNA extraction and 16S rRNA gene sequencing

Microbial genomic DNA was extracted from the stool samples using the QIAamp Fast DNA stool mini-kit (QIAGEN) and kept at − 80 °C until use. The V4 region of the 16S rRNA gene in each sample was amplified using the 515F (5′-GTGCCAGCMGCCGCGGTAA-3′) and 806R (5′-GGACTACHVGGGTWTCTAAT-3′) primer pair initially created by Caporaso et al. [56] and then sequenced using the Illumina MiSeq Personal Sequencing platform following the protocol described by Kozich et al. [57].

### Data analysis

#### Taxonomic profiling

Using QIIME 2 [58] (version 2019.1.0), the raw sequencing data was demultiplexed and sequence quality was controlled using DADA2 [59] to truncate the low quality regions of the sequences. Afterward, chimeric sequences were removed using the vsearch uchime-denovo function with default parameters [60]. The taxonomic assignation was performed using Naive Bayes classifier trained using the Greengenes taxonomy [61] from august 2013 where the sequenced were trimmed to the V4 region bound by the 515F/806R primer pair and clustered at 99% sequence identity. This taxonomic classifier is available on the QIIME 2 website (https://docs.qiime2.org/2018.11/data-resources/).

#### Analysis of alpha and beta-diversity

We studied the alpha-diversity within our cohort using the vegan [62] and phyloseq [63] package developed for the R environment for statistical computing [64]. We calculated 3 different alpha diversity measures (observed OTUs, Shannon diversity index [26] and the Simpson’s index [27]) to evaluate microbiome diversity for each patients. Observed OTUs is the number of different OTUs observed within an individual sample. Shannon diversity index quantifies the uncertainty in the prediction of the identity of an element taken at random from a sample also known as the evenness of a sample. As for the Simpson’s index, it represents the probability of two randomly picked elements of a sample being part of the same OTU. We used Student t-tests to compare alpha diversity measures between patients with and without blood in their stools. For beta-diversity, we calculated the Bray-Curtis distance [28] between all samples and plotted those distances using a Non-metric Multidimensional Scaling [65] (nMDS) ordination. We used the Bray-Curtis dissimilarity to quantify the dissimilarity between two samples using not only the presence of an element but also its abundance within the sample.

#### Differentially abundant taxa using LEfSe

For these analyses, we removed all OTUs present in less than 10% of our study population, merged OTUs at the species level and converted the read count to relative abundances. To identify differentially abundant taxa between patients with positive and negative iFOBT results we used the biomarker discovery algorithm LEfSe [29]. LEfSe identifies features with are statistically different between conditions by first using a non-parametric Kruskal Wallis sum-rank test [66] followed by a pairwise Wilcoxon rank-sum test [67] on the features that were significant in the previous step. Finally, a linear discriminant analysis [68] (LDA) model is built using features that remain significant after the two previous statistical tests to estimate the effect size.

## Data Availability

The dataset generated and analysed in the current study is publicly available in NCBI’s Sequence Read Archive (SRA) repository under the BioProject ID PRJNA577051 (http://www.ncbi.nlm.nih.gov/bioproject/577051).

## Abbreviations

CRC: Colorectal cancer
IFOBT: Immunochemical based fecal occult blood test
LDA: Linear discriminant analysis
LEfSe: Linear discriminant analysis Effect Size
NMDS: Non-metric Multidimensional Scaling
OTU: Operational taxonomic units
